# Thrombolysis-Related Intracerebral Hemorrhage and Cerebral Amyloid Angiopathy: Accumulating Evidence

**DOI:** 10.3389/fneur.2015.00099

**Published:** 2015-05-08

**Authors:** Andreas Charidimou, James A. R. Nicoll, Mark O. McCarron

**Affiliations:** ^1^Department of Neurology, Massachussetts General Hospital, Boston, MA, USA; ^2^Department of Neuropathology, University of Southampton, Southampton, UK; ^3^Department of Neurology, Altnagelvin Hospital, Londonderry, UK

**Keywords:** cerebral amyloid angiopathy, intracerebral hemorrhage, thrombolysis

Intracerebral hemorrhage (ICH) is the most feared risk of systemic thrombolysis for ST-elevated myocardial infarction, pulmonary embolism, or acute ischemic stroke. Clinical, radiological, and pathological evidence suggests that cerebral small vessel disease and, in particular, cerebral amyloid angiopathy (CAA) may contribute to or in some cases directly underpin thrombolysis-related intracerebral hemorrhage (TICH) (1). Further developments, particularly in neuroimaging, have strengthened this hypothesis, hinting at the prospect of identifying biomarkers to measure TICH risk for individual patient groups. Emerging biomarkers for CAA such as lobar cerebral microbleeds (2) may become increasingly useful for outcome endpoints in clinical trials and patient risk stratification for TICH (3).

Thrombolysis-related intracerebral hemorrhage is a complex pathophysiological process. For ischemic stroke patients, a key issue is the location of TICH, i.e., hemorrhage into the area of ischemia vs. hemorrhage in a remote non-ischemic site (occurring in about 20% of patients with symptomatic TICH). Classification of TICH has traditionally focused on clinical and radiological features (4), with less emphasis on whether different mechanisms might be implicated in TICH in remote from or within the acute infarcted region (5) or whether pathological assessment has occurred.

Coregistered Pittsburgh compound B positron emission tomography (PiB-PET) imaging has revealed that spontaneous hemorrhage hotspots preferentially occur at locations with increased amyloid β-protein burden (6). In patients treated with recombinant tissue plasminogen activator (rt-PA) for acute ischemic stroke, cerebral amyloid β-protein (as detected with PiB-PET) retention was higher in patients with parenchymal hemorrhage compared to patients without (7). Although PiB-PET has somewhat poor spatial resolution and cannot reliably resolve parenchymal and cerebrovascular amyloid β-protein, the finding is probably one of the strongest pieces of radiological evidence implicating CAA in TICH. Matrix metalloproteinase 9, a zinc-dependent endopeptidase and a marker of hemorrhagic transformation after ischemic stroke, is released from neutrophil granules by rt-PA in humans (8). Amyloid β-protein can also release and activate MMP-9 from mouse endothelial cells (9), suggesting that convergent risk factors may lead to hemorrhage.

Cerebral microbleeds identified on MRI in a lobar distribution are considered a characteristic hemorrhagic marker of a vasculopathy related to CAA (2). It has slowly emerged that multiple microbleeds might increase the risk of symptomatic ICH following thrombolysis treatment, a relationship which increases with increasing numbers of microbleeds (10, 11). In more recent studies with larger groups of ischemic stroke patients receiving intravenous thrombolysis in both European and Chinese populations, multiple cerebral microbleeds were more clearly associated with symptomatic and parenchymal hemorrhage, respectively (12, 13). Future study may provide insights into potential mechanisms, and meta-analyses may highlight the relative importance of lobar and non-lobar cerebral microbleeds in stratifying the intracerebral hemorrhagic risk from thrombolysis.

In a review in 2004 (1), we identified 10 patients with pathological investigation of TICH, 7 of whom had evidence of CAA. All of these patients had been treated for acute myocardial infarction and nine of the patients had multiple hemorrhages in a lobar distribution. With an increasing emphasis on primary percutaneous intervention for ST-elevated myocardial infarction, it may not be surprising that no further TICH cases following thrombolysis for acute myocardial infarction have been reported. However, although thrombolysis rates have increased for acute ischemic stroke patients, in an updated systematic literature search, only two further autopsy TICH cases (multiple and both hemispheres) have been reported in the stroke literature, both of whom had CAA (14). The relative lack of human pathological studies compared to neuroimaging studies hampers further developments in this area. A pathological register attached to a clinical register would enhance our understanding of TICH, particularly in the older population with acute ischemic stroke.

The known predictors of clinically significant TICH currently include age, clinical stroke severity, high blood pressure, hyperglycemia, early CT ischemic changes, large baseline diffusion lesion volume, leukoaraiosis, and cerebral microbleeds on MRI; the evidence for a role of CAA in TICH continues to accumulate.

## Author Contributions

AC conceived the idea and reviewed the literature and drafts of the paper. JN contributed to the writing, analyzed the literature, and reviewed drafts of the paper. MM wrote the first draft and reviewed drafts of the paper.

## Conflict of Interest Statement

The authors declare that the research was conducted in the absence of any commercial or financial relationships that could be construed as a potential conflict of interest.
